# The Cochrane Collaboration and the World Health Organization: new perspectives for an old relationship

**DOI:** 10.1590/S1516-31802010000200013

**Published:** 2010-03-04

**Authors:** Maria Regina Torloni

**Affiliations:** I MD, PhD. Gynecologist and obstetrician, Department of Obstetrics, and research assistant, Centro Cochrane do Brasil, Universidade Federal de São Paulo — Escola Paulista de Medicina (Unifesp-EPM), São Paulo, Brazil.

*“If you want to go fast, go alone. If you want to go far, go together.”*

Dr. Margaret Chan, WHO Director General, Dec 2007.

The Cochrane Collaboration and the World Health Organization (WHO) have a long history of productive collaboration. Over the years, many members of the Cochrane Collaboration have been actively involved in producing systematic reviews and have contributed towards developing guidelines for various WHO departments. However, these collaborations have always been based on informal personal contacts between individuals in the two organizations. Although these informal collaborations will not be precluded in any way in the future, there is a clear need to improve communication and optimize the links between the two organizations. To maximize synergies and improve outcomes, WHO set up a formal meeting between representatives of the two organizations that was held in Singapore on October 9-10, 2009, immediately preceding the 17^th^ Cochrane Colloquium.

A selected number of participants from each organization discussed the functions and principles of each entity, and how they could develop a strategy for formal collaboration. The main aim of this meeting, which I had the privilege of attending, was to determine how a formal partnership between WHO and the Cochrane Collaboration could be established to improve the connection between the providers and implementers of research evidence, particularly in low and middle-income countries. A presentation summarizing the main points of this meeting can be seen at: http://www.cochrane.org/multimedia/colloquium_2009/apreso/plenary1_tovey_ghersi.htm.

A plan for collaboration was established and the Cochrane Collaboration was invited to apply for formal NGO (non-governmental organization) status with WHO. Once approved, this official NGO status would give the Cochrane Collaboration a seat in the World Health Assembly. This assembly, which takes place in Geneva in May of each year, is the supreme decision-making body for WHO. It is attended by delegates from all 193 member states and establishes the priorities and policies for WHO. This new status could potentially increase the impact of Cochrane reviews, particularly in low and middle-income countries. Additionally, the Cochrane Collaboration would have the opportunity to contribute towards health policy and guideline development and to participate in joint advocacy for evidence-informed decision-making and resourcing throughout the world.

Communication between the two organizations is an essential part of this collaboration, and therefore it was decided that WHO Regional and Country offices would make efforts to improve the links with existing Cochrane Centers in their regions. The following additional initial tasks were agreed upon by both partners: capacity-building for reviews in developing countries (an activity that could use the Brazilian Cochrane Center's successful experiences as a model), priority review production and a continuing dialog on different methodologies, along with systematic reviews of randomized clinical trials.

The prospects for this Cochrane-WHO collaboration are excellent for both institutions and, more importantly, for improvement and dissemination of evidence-based healthcare for the populations of low and middle-income countries throughout the world.

